# 국내 생체 간이식 공여자의 수술 후 적응에 관한 통합적 고찰

**DOI:** 10.7586/jkbns.26.017

**Published:** 2026-05-26

**Authors:** Gyeong-Ju An

**Affiliations:** Department of Nursing, Cheongju University, Cheongju, Korea; 청주대학교 간호학과

**Keywords:** Liver transplantation, Tissue donors, Adaptation, physiological, Adaptation, psychological, Review, 간 이식, 공여자, 생리적 적응, 심리적 적응, 종설

## Abstract

**Purpose:**

This study aimed to examine postoperative adaptation among living liver donors in South Korea through an integrative review.

**Methods:**

This integrative review followed the five-stage framework proposed by Whittemore and Knafl. A comprehensive search of peer-reviewed publications published from 1994 to 2025 was conducted across seven electronic databases using keywords such as “liver transplantation,” “living donor,” and “Korea.” The initial search identified 287 records, and 11 studies were included after screening, eligibility assessment, and quality appraisal.

**Results:**

The 11 included studies consisted of descriptive studies (n = 10) and one qualitative study (n = 1). The integrated findings on postoperative adaptation among living liver donors were as follows. First, donors experienced physical adaptation challenges because of discrepancies between their preoperative expectations regarding pain, recovery speed, and complications and their actual postoperative experiences. Second, donors faced psychosocial burdens related to surgical scarring, uncertainty about the future, and financial strain. Third, donors reported strengthened family relationships and increased life satisfaction after transplantation. However, existing instruments for measuring health-related quality of life lack the sensitivity needed to capture donor-specific experiences.

**Conclusion:**

These findings highlight the urgent need to develop counseling programs that provide realistic information about the physical adaptation of living liver donors.

## 서론

### 1. 연구의 필요성

간이식은 현재 유일한 말기 간질환의 치료방법이며 간을 뇌사자로부터 기증받는 뇌사 간이식(deceased donor liver transplantation)과 건강한 성인으로부터 기증받는 생체 간이식(living donor liver transplantation)으로 구분된다. 뇌사 간이식 수혜자의 1년 생존율이 75.1%인데 비해 생체 간이식 수혜자의 생존율은 90.1%로 더 높고[1] 뇌사자 기증이 적은 문화적 영향으로 인해[2] 2024년 통계에 따르면 국내 간이식의 72.9%를 생체 간이식이 차지하고 있다[1]. 국립장기조직혈액관리원의 2024년 자료에 의하면 생체 간이식 920건의 공여자 중 수혜자의 자녀인 경우 575건(62.5%), 배우자인 경우 149건(16.2%)으로[1] 대부분 가족의 장기에 의존하는 혈연관계 공여라는 특징이 있다. 따라서 가족 내 공여가 대부분인 국내 현실에서 생체 간이식 공여자가 다시 이전의 일상생활로 돌아가는 수술 후 적응을 돕는 것은 건강한 가족을 이루기 위해서 사회적 차원에서도 중요한 문제이다.

이식수술 후 적응이란 현재의 치료 단계를 수용하고 주어진 상황에서 신체적, 심리적, 사회적으로 안녕을 유지하는 것이므로, 의료진은 생체 간이식 공여자의 수술 후 안녕을 증진하기 위해 신체적, 심리적, 사회적 적응과정을 적극적으로 지원하는 것이 필요하다. 하지만 생체 간이식에서 공여자가 매우 중요한 위치에 있음에도 불구하고 생체 간이식 공여자의 수술 후 적응과정에 관한 관심은 간이식 수혜자에 비해 상대적으로 매우 부족하였다[3]. 일반적으로 이식 외과의는 생체 간이식 후 공여자의 남아있는 간이 1년 안에 다시 재생된다고 기대하기 때문에[4] 생체 간이식 공여자 보다는 간이식 수혜자의 건강상태와 생존율을 더 중대하게 여기므로, 수혜자와 관련된 연구들이 많이 수행된 것에 비해 생체 간이식 공여자의 신체적, 심리사회적 적응에 관해서는 간과되어왔다[2-5].

하지만 국외에서는 생체 간이식 공여자에 대한 관심이 국내보다 높아 2015년에 생체 간이식 공여자 코호트 연구를 최초로 대규모로 수행하여, 생체 간이식 공여자의 안녕과 건강관련 삶의 질(health-related quality of life, HRQOL)이 일반인들에 비해 높게 나타났고 90%의 생체 간이식 공여자들이 심리적 성장을 경험하였다는 긍정적인 결과를 보고하였다[4,5]. 또한 국외 생체 간이식 공여자의 삶의 질에 관한 19개 연구를 대상으로 한 체계적 문헌고찰 연구에서는 생체 간이식 공여자의 80%~93%에서 삶의 질 수준이 이식수술 후 6개월~1년 안에 수술 전 수준으로 회복된다고 보고한 바 있다[3,6]. 국내에서 생체 간이식 공여자를 대상으로 이루어진 일부 연구에서는 간이식 수술 후 3개월까지 이식수술 전에 비해 삶의 질이 더 낮은 수준으로 저하되었으며 간 공여 후 3개월에 21명(72.4%)이 직장에 복귀하였다고 보고하였으나[7], 세계 국가별로 생체 간이식 공여자 선정과정, 의료체계, 사회적 분위기, 가족문화 등이 다르기 때문에 생체 간이식 공여자의 수술 후 신체적, 심리사회적 적응과정은 국내와 국외 간 차이가 있을 수 있다.

국외에서는 생체 간이식 공여자의 신체적, 심리사회적 적응에 관한 연구가 많이 이루어졌기 때문에[8] 이를 토대로 생체 간이식 공여자의 우울에 관해 메타분석을 한 연구[9]와 삶의 질에 관한 문헌고찰 연구[3]가 발표되어 생체 간이식 공여자의 심리적 적응과정을 자세히 살펴볼 수 있었다. 하지만 국내에서는 생체 간이식 공여자의 수술 후 삶의 질에 관한 연구가 2004년 처음 발표된 이후[10], 생체 간이식 공여자 대상으로 수술 후 신체적, 심리사회적 적응에 관한 연구가 소수 발표되었으나 이러한 관련 연구결과들을 통합적으로 고찰하여 국내 생체 간이식 공여자의 신체적, 심리사회적 적응과정을 전체적으로 파악한 연구는 아직 이루어지지 않았다.

생체 간이식 공여 의사가 있는 공여자가 나타나게 되면 간이식 절차를 관리하는 장기이식코디네이터가 공여자에게 현실적으로 필요한 정보제공을 통해 상담과 교육을 실시하는데, 간이식 공여를 고려하고 있는 생체 간이식 공여자 입장에서는 수술 후 시간의 경과에 따라 신체적, 심리사회적으로 어떻게 적응해 나가는지에 대한 구체적인 정보가 절실하게 필요하다[11]. 이 과정에서 생체 간이식 공여자가 어떻게 신체적, 심리사회적으로 실제 적응해 나가는지에 관해 국내 연구들을 분석한 통합적 고찰 연구결과를 바탕으로 상담한다면 생체 간이식 공여자의 수술 후 적응에 도움이 될 수 있을 것이다.

따라서 본 연구에서는 국내에서 이루어진 생체 간이식 공여자의 신체적, 심리사회적 상태에 관한 연구들을 통해 국내 생체 간이식 공여자가 신체적, 심리사회적으로 어떻게 적응해 가는지를 통합적으로 고찰함으로써 향후 국내 생체 간이식 기증을 고려하는 공여자 상담과 교육 프로그램을 개발할 때에 중요한 기초자료를 제공하고자 한다.

### 2. 연구의 목적

본 연구의 목적은 생체 간이식 공여자가 수술 후 신체적, 심리사회적으로 어떻게 적응하는지를 통합적 문헌고찰을 통해 분석함으로써 생체 간이식 공여자의 수술 후 적응과정을 파악하여 향후 생체 간이식 공여자를 위한 상담 및 교육중재 개발 시 기초자료를 제공하기 위함이다.

구체적인 연구 목적은 다음과 같다.

첫째, 선정된 논문을 통해 생체 간이식 공여자의 신체적 적응을 확인한다.

둘째, 선정된 논문에 나타난 생체 간이식 공여자의 심리사회적 적응을 확인한다.

## 연구 방법

### 1. 연구 설계

본 연구는 Whittemore and Knafl[12]이 제시한 통합적 고찰 방법에 따라 국내 생체 간이식 공여자의 수술 후 신체적·심리사회적 적응 관련 연구를 분석·합성한 통합적 문헌고찰 연구이다.

### 2. 연구 대상

본 연구 대상의 선정 기준은 국내에서 생체 간이식 공여자를 대상으로 수행된 연구 중에서 이식수술 후 신체적, 심리사회적 상태에 관한 질적, 양적 논문으로 하였다. 구체적인 선정 기준은 국내에서 생체 간이식 수술이 성공한 시기가 1994년임을 고려하여 1994년 1월부터 2025년 12월까지 동료심사 제도를 갖춘 국내·외 전문 학술지에 출판된 논문 중에서 생체 간이식 공여자의 수술 후 신체적, 심리사회적 상태를 설문조사 또는 인터뷰를 통해 자료를 수집하였거나 임상검사로 측정한 논문들로 선정하였다.

### 3. 연구 절차

본 연구에서 이용한 통합적 고찰이라는 연구방법은 특정한 주제에 대해 포괄적인 이해를 제공하기 위해 조사 연구, 실험 연구, 질적 연구 등 다양한 연구방법으로 수행된 관련 주제 연구들을 모두 포함하여 분석 후 범주화하고 합성함으로써 통합적으로 고찰하는 방법이다[12]. 본 연구는 Whittemore and Knafl [12]의 연구에 제시되어 있는 통합적 고찰 방법 5단계에 따라 연구를 진행하였다.

#### 1) 1단계: 문제 확인(Problem identification stage)

일반적으로 국내 간이식 사례가 많은 병원에서는 생체 간이식 공여자에게 이식 후 적응과정을 설명할 때 절제된 간은 이식수술 후 1주일 동안 전체 간의 약 60%, 3개월 동안 약 80%가 재생되므로, 간이식 수술 후 한 달쯤이면 간 기능이 정상화되며, 수술 후 4~6주 지나면 집안일이나 간단한 서류업무가 가능하고[13], 간이식 수술 후 2개월이면 수술 전과 같이 정상으로 된다고 말한다[2,14]. 그러나 의료진의 이러한 설명과 달리 실제 간이식 공여자들은 신체적으로 정상이 되는 데에 6개월~1년 이상 걸리고[2], 1년이 지나도 이식수술 이전과 동일한 정상상태로 돌아가지 못한다고 보고한 연구들이 있다[14]. 그러므로 본 연구에서의 연구문제는 ‘국내 생체 간이식 공여자는 이식수술 후 어떻게 신체적, 심리사회적으로 적응하는가?’ 이다.

#### 2) 2단계: 문헌 검색(Literature search stage)

**Figure 1.**
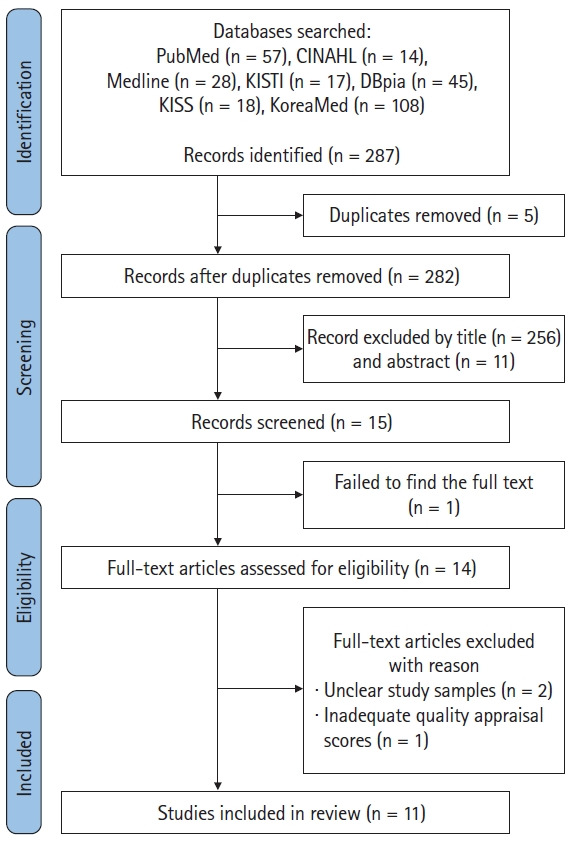
PRISMA flow diagram of the articles retrieved and selection process. CINAHL = Cumulative Index to Nursing and Allied Health Literature; KISTI = Korea Institute of Science and Technology Information; KISS = Korean Studies Information Service System.

본 연구의 문헌 검색은 2025년 12월 1일부터 12월 22일까지 시행되었다. 문헌 검색 및 선정 과정은 연구자가 PRISMA (Preferred Reporting Items for Systematic Reviews and Meta-Analyses) flow diagram을 참고하여 문헌 선정의 일관성과 분석의 재현 가능성을 확보하는 데에 초점을 두어 4단계 절차인 식별(Identification), 검사(Screening), 적격성 평가(Eligibility), 포함(Inclusion)의 단계별로 체계적이고 엄격한 절차로 수행하였으며 Figure 1에 제시하였다. 검색 문헌의 출판년도는 국내에서 생체 간이식이 시작된 1994년 1월부터 2025년 12월로 제한하여 국내·외 학술지에 발표된 연구문헌을 검색하고 자료를 수집하였다.

##### (1) 선정 기준

- 양적 연구인 경우 국내 생체 간이식 공여자의 신체적, 심리사회적 변수를 측정하여 통계적으로 분석한 연구

- 질적 연구인 경우 국내 생체 간이식 공여자와의 인터뷰 내용을 통해 경험을 탐색한 연구

- 한국어와 영어로 출판된 논문

- 동료 심사 학술지에 게재된 논문

- 전문(full-text) 확보가 가능한 논문

##### (2) 제외 기준

- 인간을 대상으로 하지 않은 논문

- 국외 생체 간이식 공여자를 대상으로 이루어진 논문

- 신장 등 다른 장기 공여자를 포함한 경우

- 공여자의 간 절제술 술기의 효과에 초점을 둔 경우

- 종설이나 학위논문

첫 번째 단계로, 국내외 논문 검색을 위해 한국과학기술정보연구원(KISTI), 한국학술정보(Korean Studies Information Service System, KISS), 학술데이터베이스서비스(Data Base Periodical Information Academic, DBpia), MEDLINE (Medical Literature Analysis and Retrieval System Online), PubMed, CINAHL (Cumulative Index to Nursing and Allied Health Literature), KoreaMed 검색 데이터베이스를 이용하여 한글 검색어는 대상자를 ‘기증자’ OR ‘공여자’로, 중재는 ‘간 이식’ OR ‘간이식’을 불리언 연산자 AND를 적용한 조합으로 ‘(기증자 OR 공여자) AND (간이식 OR 간 이식)’ 검색식을 입력하였다. 영어 검색어는 대상자 ‘donor’ ‘tissue donors [MeSH terms]’와 중재 ‘liver’ ‘transplant’ ‘transplantation [MeSH terms]’을 불리언 연산자를 적용하여 ‘(donors OR tissue donors [MeSH terms]) AND (liver OR transplant OR transplantation [MeSH terms]) AND (korea OR korean)’ 검색식을 영어 검색데이터베이스에 입력하여 검색을 시행하였다. 검색 결과, KISTI 17편, KISS 18편, DBpia 45편, PubMed 57편, MEDLINE 28편, CINAHL 14편, KoreaMed 108편으로 총 287편으로 나타났다. 두 번째 단계로, RefWorks를 이용하여 미리 검색 데이터베이스를 통해 입력된 논문들에서 중복검사를 실시하여 제목, 저자, 연도가 일치하는 중복문헌 5편을 제거하여 282편이 되었다. 세 번째 단계에서는 논문의 제목과 초록을 검토하면서 선정 기준에 부합하지 않는 256편과 11편을 제외하여 15편의 논문이 선정되었다. 네 번째 단계에서는 15편 중 전문 확보가 불가능한 1편을 제외하여 14편이 되었다. 다섯 번째 단계에서는 14편 논문의 전문을 검토하면서 연구대상자가 존재하나 대상자 정보가 명확히 제시되지 않은 논문 1편과 실제 간 공여를 하지 않은 잠재적 공여자를 포함한 논문 1편을 배제시켜 최종 검색단계에서 총 12편의 논문을 확인하였다.

#### 3) 3단계: 자료 평가(Data evaluation stage)

통합적 고찰 방법론에서는 자료들이 복합적이기 때문에 자료의 질을 평가할 때에 표준지침이 제시되지 않았으나[12], 본 연구에서는 검색된 논문의 질적 수준을 평가하기 위해 타당성과 신뢰성이 검증된 혼합방법평가도구(Mixed Method Appraisal Tool, MMAT)를 이용하여 연구의 편향성 위험(Risk of bias)을 평가하였다[15]. MMAT는 질적 연구, 양적 서술 연구, 비무작위 정량 연구, 무작위 대조군 연구, 혼합 연구의 다섯 가지 설계 유형에 따라 평가 항목을 구분하여 적용할 수 있는 통합적 평가 도구로, 다양한 연구 설계를 포함하는 본 통합적 고찰의 특성에 적합한 도구이다. 또한, 연구자 단독 평가에서 발생할 수 있는 주관성을 줄이기 위해, 연구자가 MMAT 기준에 따라 질 평가를 수행한 후 간호학 교수 1인의 자문을 받아 평가 결과를 검토하여 평가의 신뢰도를 확보하려고 노력하였다.

각 연구유형에 따른 MMAT에 의한 질 평가 결과는 Table 1 [10,16-26]에 제시하였다.

모든 연구들의 공통 평가문항인 연구문제의 명확성(S1)과 수집된 데이터의 타당성(S2) 항목에서 전체 12편 논문이 적합하게 평가되었다. 질적 연구(1편)는 연구질문의 적절성(1.1), 자료수집 방법의 적절성(1.2), 결과 분석의 적절성(1.3), 결과 해석의 적절성(1.4), 질적 자료수집과 분석 및 해석의 일관성(1.5)에 대해 충족한 것으로 평가되었다. 양적 서술연구(11편)는 표본 추출 방법의 적절성(4.1), 도구의 적절성(4.3), 무응답 편향 위험(4.4) 항목에서는 11편 모두 기준을 충족하였으나, 표본의 대표성(4.2)과 통계분석의 적절성(4.5) 항목에서 논문 1편이 ‘아니오’로 평가되었다. 본 연구에서는 선행연구[15]의 권고에 따라 점수를 합산하지 않고, 각 세부 항목의 충족 여부를 바탕으로 연구의 질을 비판적으로 검토하여 You 등의 논문[21]은 표본수가 적은 상태에서 여러 개의 독립변수를 다중회귀분석에 투입하여 통계적 검증력이 떨어지므로 질적 수준이 충족되지 않았으므로 질 평가에서 1편을 제외시켜 최종적으로 통합적 고찰에 포함된 논문은 11편이 되었다.

#### 4) 4단계: 자료 분석(Data analysis stage)

본 연구자는 이미 통합적 고찰연구를 수차례 수행한 경험이 있기 때문에 이를 바탕으로 자료 분석 단계에서 11편의 논문 원문 전체를 읽으면서 다음과 같은 순서로 진행하였다. 첫째, 각 문헌을 연구자, 연도, 연구방법, 주요결과 등을 요약하면서 연구결과에서 나타나는 문장 중 반복되거나 의미 있는 구절을 추출하였다. 둘째, 의미 있는 결과 구절들을 신체적 영역과 심리사회적 영역으로 구분하여 유사한 자료별로 분류하면서 표로 만드는 매트릭스 작업을 하였다. 이 과정에서 문헌들의 결과에서 공통점이나 차이점을 파악함으로써 유형화하였다. 셋째, 비슷한 유형들을 범주화하여 해당 내용들을 포함할 수 있는 주제를 도출함으로써 연구결과들을 통합하였다. 넷째, 연구문제에 대한 통합적인 답변이 되는지 도출된 주제에 대해 확인하였다[12].

#### 5) 5단계: 자료 제시(Review presentation)

5단계에서는 선정된 문헌들을 분석한 후 범주화하여 통합적으로 고찰한 결과를 제시한다. 이때 통합적 결론을 뒷받침할 일차적 자료 결과와 함께 제시함으로써 새로운 현상에 대한 이해를 돕게 된다. 본 연구에서는 국내 생체 간이식 공여자의 수술 후 적응과정을 조사하거나 탐색한 국내·외 연구를 고찰하고 그 결과에 대한 논리적 근거와 이해를 증진시키기 위해 일차 문헌의 주요 내용을 표로 제시하였다(Table 2).

### 4. 윤리적 고려

본 연구는 이미 출판되어 공개된 연구논문들을 대상으로 하는 문헌고찰 연구이기 때문에 청주대학교 윤리위원회의 심의면제 승인을 받았다(IRB No: 1041107-202602-HR-001-01).

## 연구 결과

### 1. 선정된 연구의 일반적 특성

본 연구에서 최종적으로 통합적 고찰에 포함된 논문은 11편이었다(Appendix 1). 논문들을 발표 연도별로 분류했을 때 2001-2010년 6편[10,16-20], 2011-2022년 5편[22-26]으로 나타났다. 연구 설계는 서술적 연구가 10편이었고[10,16-20,22-24,26] 질적 연구 1편[25]이었으며, 연구방법은 전향적 조사 2편[10,23], 후향적 의무기록 조사 4편[16,17,22,24], 횡단적 조사 4편[18-20,26], 인터뷰 1편[25]이었다. 전향적 조사인 경우에 추적기간이 평균 12개월[10], 19개월[23]인 반면에 후향적 의무기록 조사인 경우에는 최장 966일[16]~10년[22]으로 추적기간이 길었다. 연구대상자 수는 질적 연구에서 8명[25]이었고 양적 연구에서는 최소 64명[20]~최대 832명[23] 범위였다.

### 2. 생체 간이식 공여자의 특성

선정된 11개의 논문에서 생체 간이식 공여자의 성별 분포는 남성의 비율이 59.3%[24]~80.8%[16]로 여성의 비율 19.2%[16]~43.5%[26] 보다 많았고, 생체 간이식 공여자들의 평균 연령은 26.4세[25]~37.9세[26]의 범위로 20~30대가 많은 것으로 나타났다. 연구대상인 생체 간이식 공여자들은 간이식 수술을 한지 평균 23.1개월[25]~35.8개월[19] 경과된 것으로 나타났고, 간이식 수술 후 1~3년 이내가 38.7%[26], 39.0%[19]로 가장 많았다.

생체 간이식 공여자의 간 절제술에 대해 제시한 연구는 7편[10,16,17,20,22-24]이었고, 좌측 간절제술 7.1%[23]~48.1%[16], 우측 간절제술 51.9%[16]~92.9%[23]로 우측 간절제술을 더 많이 하는 것으로 나타났다. 그중 Lee 등[22]의 연구는 연구대상자를 우측 간절제술 공여자로 제한하여 진행하였기 때문에 우측 간절제술이 100.0%였다. 또한 간절제술 시 복강경을 이용한 경우 78.0%, 개복수술을 한 경우(22.0%)로 나타났다[24].

간이식 수혜자와 생체 간이식 공여자의 관계에 대해 살펴보면 수혜자의 자녀가 전체 생체 간이식 공여자의 33.7%[16]~71.0%[26]를 차지하여 가장 높게 나타났고, 간절제술에 따라 구분한 연구에서는 좌측 간절제술 생체 간이식 공여자의 40.7% 우측 간절제술 생체 간이식 공여자의 72.2%가 수혜자의 자녀인 것으로 나타났다[23]. 생체 간이식 공여자가 배우자인 경우 6.3%[20]~19.8%[24]였고, 형제자매인 경우 8.9%[26]~18.3%[16]로 나타났다. 특히 친척이 아닌 경우에는 2.9%[19], 8.7%[10], 14.4%[16]으로 나타났고, 순수한 타인의 기증임을 표기한 연구는 1편[20]이었다 (Table 2).

### 3. 생체 간이식 공여자의 수술 후 적응

#### 1) 신체적 적응: 회복 과정에 대한 기대와 실제 경험의 차이

##### (1) 예상보다 극심한 통증

생체 간이식 공여자가 이식수술 후 3일까지 시각적 통증 척도(visual analog scale)로 측정한 통증의 수준은 5~8점 사이로 나타나 간암절제술 환자의 수술 후 1~2일에 동일한 척도로 측정한 2~3점 보다 높았다[20]. 질적 연구에서도 ‘죽을 만큼 힘들고 아팠던 극심한 수술 통증’이라는 진술을 하였으며[25], 생체 간이식 공여자들의 34.7%가 이식수술 전에 예상했던 것보다 통증이 심했다고 응답하였다[26]. 또한 생체 간을 비자발적으로 공여한 공여자의 통증 점수가 자발적으로 공여한 공여자의 통증 점수보다 유의하게 높은 것으로 나타났다[20]. 생체 간이식 공여자들은 수술 직후 통증 경감을 위해 자가조절진통(patient-controlled analgesia, PCA) 장치를 적용하지만, 추가적으로 진통제를 1~2가지 더 사용한 생체 간이식 공여자도 41.8%나 될 정도로 심한 통증을 경험하였고, 구역질과 어지럼증 같은 PCA 관련 부작용을 경험한 경우도 59.3%로 나타났다[24]. 생체 간이식 공여자의 진술문 중 간절제술을 위해 개복수술로 가슴부터 배꼽 위까지 복근을 갈라놓아 살을 양쪽에서 뜯는 것처럼 아팠다는 진술이 있었으나[25], 생체 간이식 공여자의 간절제술을 복강경수술과 개복수술로 했을 경우 통증의 차이를 비교한 연구에서는 각각 3.45점, 3.56점으로 복강경수술 후 통증이 약간 낮게 나타났으나 통계적으로 유의한 차이가 없었다[24].

##### (2) 기대보다 느린 회복속도

본 연구에서 나타난 생체 간이식 공여자들의 평균 재원기간은 평균 9.24일[24]~18.0일[16]이었고, 20일 이상 입원한 경우도 17.6%[18]로 나타나기도 하였다. 생체 간이식 공여자의 22.6%가 자신들의 기대와 달리 회복되는 속도가 느렸다고 응답했으며, 9.7%는 재원기간이 길어졌다고 응답하였다[26]. 간절제술 수술방법에 따른 회복속도의 차이를 확인한 결과에서는 복강경수술 공여자의 재원기간이 8.9 ± 2.10일로 나타나 개복수술 공여자의 재원기간 10.4 ± 3.46일 보다 재원기간이 유의하게 짧아 복강경수술로 간절제술을 실시하는 것이 회복속도가 빠르다고 하였다[24]. 또한 간절제술 유형에 따라 조사한 연구에서는 좌엽절제술을 받은 공여자가 우엽절제술을 받은 공여자에 비해 간기능검사가 빠르게 정상으로 회복되었고 중환자실 체류기간도 짧았으나 퇴원까지의 재원기간은 유의한 차이가 없이 비슷한 것으로 나타났다[16].

생체 간이식 공여자의 적응 속도가 느리면 직장으로의 업무 복귀도 지연되는데, 본 연구에서 선정된 논문 중 Kim 등[19]의 연구에서는 이식수술 후 3개월 안에 공여자의 46.5%가 직장으로 복귀하였고 96.9%가 1년 내에 직장 업무로 복귀한 것으로 나타났으며, 이들 중 자신이 하는 일에 제약을 느끼지 않는다고 응답한 경우가 75.0%였다. Yoo 등[10]의 연구에서는 수술 후 3개월 내에 직장으로 복귀한 경우가 76.0%로 나타났고 6개월까지 업무 복귀를 한 공여자는 89%로 나타났으나, Lee 등[23]의 연구에서는 이식수술후 공여자 100%가 6개월 이내에 정상생활로 복귀하였다고 하였다. 그러나 질적 연구에서는 ‘체력이 안되고 힘들어서 회사에서 멀쩡한 척 일하고 집에 와서 바로 잤다’는 진술처럼[25] 이식수술 후 완전히 적응이 되지 않은 채 직장생활을 하는 경우가 있었다. 또한 간이식 관리에 관한 생체 간이식 공여자의 지식 정도와 직장복귀 소요기간이 유의한 관계가 있는 것으로 나타났다[19].

생체 간이식 공여자의 간 기능에 영향을 미칠 수 있는 알코올 섭취에 대해 조사한 연구는 1편[23]이었고 이식수술 후 생체 간이식 공여자가 알코올을 섭취하는 경우는 45.6%로 나타났으며 그중 3.8%는 습관성 음주라고 응답하였다[23].

##### (3) 기대하지 않았던 합병증 발생

선정된 논문들에서 나타난 이식수술 후 합병증 발생률은 다양하게 나타났다. 수술 전에 기대했던 것과 달리 공여자의 7.3%가 실제로 합병증이 악화되었다고 응답하였고[26], 이식수술 후 4개월 이내 합병증 발생률 6.5%[10]로 낮은 합병증 발생률이 나타난 반면 재원기간 중 합병증 발생률 44.0%[24], 수술 후 8개월까지 합병증 발생률 46.1%[22], 수술 후 평균 2년 정도의 기간 중 합병증 발생률 48.3%[26]로 높게 보고한 연구들이 있었다. 국내 15개 병원에서 대규모 코호트 연구로 진행한 연구에서는 합병증 발생률이 평균 9.3%로 나타났고 그중 담도계 합병증 발생률 1.7%, 주요 합병증 발생률 1.9%로 나타났다[23]. 합병증을 총 5단계로 분류하는 클라비안 딘도 분류(Clavien-Dindo classification)를 이용하여 합병증을 조사한 연구는 3편[22,23,26]이었다.

생체 간이식 공여자의 수술 유형에 따라 좌엽절제술을 받은 공여자의 합병증 발생률은 4.0%인 반면에 우엽절제술을 받은 공여자의 합병증 발생률은 13.0%로 나타나 우엽절제술의 합병증 발생률이 높았음을 보여주었고 주로 합병증은 담즙 누출, 출혈 등이었다[16]. 또한, 수술 방법에 따라 구분한 연구에서 개복수술을 한 경우의 합병증 발생률은 55.0%, 복강경으로 수술한 경우의 합병증 발생률은 40.9%로 나타나 개복수술시 합병증 발생률이 높았으며 주로 나타난 증상은 복강내 저류였다[24]. 또 다른 연구에서는 병원이나 약국을 방문할 정도의 증상을 경험한 경우가 대상자의 23.9%로 나타났고 주로 소화계 증상으로 나타났으며[10], 수술 후 집에서 발생하는 합병증 증상 등으로 인해 공여자들의 교육요구도 조사에서 가장 높게 나타난 항목이 ‘간 공여수술 후 관리‘였다[19].

#### 2) 심리사회적 적응: 수술 후 생긴 부담감

##### (1) 수술 흉터에 대한 스트레스

생체 간이식 공여자의 59.5%가 간절제술로 인해 생긴 수술 흉터로 심리적 스트레스를 받고 있다고 하였고 그중 8.7%는 흉터로 인해 항상 스트레스를 받는다고 하여 부정적인 신체상이 형성됨을 보여주었다[10]. 질적 연구에서도 생체 간이식 공여자들은 수술 흉터에 대한 사람들의 불편한 시선으로 대중목욕탕이나 워터파크 등의 방문을 피하게 되었고 일부 대상자는 흉터치료전문병원을 알아보는 등 흉터 제거에 대한 마음고생을 하며 이식수술 흉터로 인해 큰 스트레스를 경험하고 있었다[25].

##### (2) 불확실한 미래와 재정적 어려움

생체 간이식 공여자가 지각한 경제적 상태에 대해 조사한 연구는 2편이었으며 불충분하다고 응답한 공여자가 37.0%[10]와 41.9%[19]로 나타났고 그중 8.8%[19]는 경제적으로 극심한 재정적 어려움을 느낀다고 응답하였다. 또한 질적 연구에서는 기증 전부터 각종 검사가 의료보험 적용이 되지 않기 때문에 비용을 100% 내야 되므로 경제적으로 힘들었다고 하였다[25]. 그 외의 연구들은 월 소득을 금액에 따라 구간별로 단순 조사하였기 때문에 공여자가 경제상태를 어떻게 주관적으로 인식하는지에 대해서는 확인이 어려웠다[18,20,26].

또한 생체 간이식 공여자들의 대다수가 청년층이기 때문에 간 공여로 인해 학업이나 사회생활을 늦추게 되어 경제 활동에 영향을 받게 되었고, 간 공여를 이유로 취업에서 불이익을 경험해서 새로운 직장에서는 간 공여 사실을 숨기고 취업했다고 진술하였다[25].

#### 3) 심리사회적 적응: 가족관계와 삶에 대한 만족

##### (1) 깊어진 가족관계

생체 간이식 공여자들은 간 기증을 계기로 서로의 건강을 챙기며 이전보다 끈끈한 가족 유대가 형성되었다고 하며 가정의 평안을 되찾고 더 화목해졌다고 진술하였다[25]. 특히 간이식 수혜자와 의 친밀도가 간이식 수술 전 8.96점에서 수술 후 9.28점으로 상승하였음을 보여주었다[19].

##### (2) 이식에 대한 만족

HRQOL을 측정한 연구에서 한국인 성인의 평균 점수보다 생체 간이식 공여자의 HRQOL이 신체적, 정신적 영역 모두 높게 나타났으며[26], 수술 후 자신의 전반적 삶의 만족도에 대해 생체 간이식 공여자의 87.0%가 만족한다고 하였다[10]. 간을 공여하기로 결정한 것에 만족하는 경우는 96.8%[26], 간 공여를 후회하지 않는다는 경우가 93.5%[10]로 나타나 생체 간이식 공여에 대한 만족도가 높게 나타났고, 주변의 배려와 효자라는 칭송과 격려를 받으면서 덕분에 힘을 얻어 잘 견디어 내었다고 하였다[25].

## 논의

본 연구는 1994년 1월부터 2025년 12월까지 발행된 논문 중 국내 생체 간이식 공여자의 수술 후 신체적, 심리사회적 적응에 대해 조사한 관련 논문 11편을 통합적 고찰로 분석하여, 향후 증가하는 생체 간이식 수술 후 공여자의 실제적인 신체적, 심리사회적 적응을 확인함으로써 생체 간이식 공여자의 상담과 교육시 도움이 되는 기초자료를 제공하고자 시도되었다.

본 연구결과에서 선정된 11편의 논문에서 국내 생체 간이식 공여자의 수술 후 적응에 관해 처음 연구가 발표된 것은 2004년 Yoo 등[10]에 의해 생체 부분 간이식 공여자의 삶의 질에 관해 조사한 것으로 나타났는데, 소아 부분 간이식을 1990년부터 시작한 일본에서는 1993년에 생체 부분 간이식 공여자의 삶의 질에 관한 연구가[27] 발표되어 한국보다 생체 간이식 공여자에 대한 관심이 일찍부터 높았음을 알 수 있었다. 특히 Morimoto et al.[27]의 연구에서는 소아 생체 부분 간이식 공여자인 부모를 대상으로 삶의 질을 확인하였지만, 본 연구에서 연구대상자 중 소아 생체 부분 간이식 공여자가 일부 포함된 논문은 7편[10,16,19,20,23,24,26]이었다. 소아 생체 부분 간이식의 경우 대부분 공여자는 부모인 경우가 많고 간 절제량도 성인 생체 간이식 공여자에 비해 적게 절제하기 때문에 적응과정도 다를 것으로 예상되므로 성인 생체 간이식 공여자와는 다른 시각에서 접근해야 한다. 그러나 국내에서 자신의 자녀에게 간 일부를 공여한 소아 생체 부분 간이식 공여자만을 대상으로 수술 후 적응을 조사한 연구는 찾아보기 어려웠고 따라서 향후 이와 관련된 연구가 필요하다고 생각한다.

국내 생체 간이식 공여자 중 가장 많은 비율을 차지하는 것은 20~30대 남성이었으며, 이러한 결과는 서구 국가들에서 생체 간이식 공여자가 남성보다 여성이 많은 것에 비해 한국, 이집트, 사우디 아라비아에서는 남성 공여자의 비율이 70% 이상을 차지하는 결과를 보고한 선행연구 결과와 일치한다[28]. 국내 생체 간이식에서 남성 공여자가 더 선호되는 이유는 대부분의 수혜자인 남성에게 주기 위해서는 충분한 양의 간용적을 가졌기 때문이며 이식수술 후 생체 간이식 공여자에게 남는 흉터가 젊은 여성의 경우에 큰 부담이 되기 때문으로 유추할 수 있다. 선정된 연구에서 간이식 공여자들의 90% 이상이 자식, 배우자, 형제, 부모 등 가족관계에 있다는 특징은 신장이식에서도 공여자의 약 95%가 가족 간 이식이라는 연구결과와 비슷하다[29]. 즉, 가족에 대한 도의와 효를 강조하는 유교문화의 특성이 남아있는 한국 등 일부 아시아권에서 혈연간 장기이식이 높은 비율로 나타나기 때문에[2] 간이식 수혜자와 공여자가 공존하는 가족의 특성으로 그들을 간호해야 하는 가족원의 신체적, 경제적 부담이 가중된다. 이러한 결과는 신장이식 수혜자와 공여자를 가진 가족에서도 동일하게 나타나는데 가족 내 신장이식 수혜자가 있을 경우 신장 공여자에 대한 고마움과 함께 흉터자국을 보면 죄책감을 느끼기도 하며, 이러한 요인들이 가족 갈등으로 발전하고, 가족 스트레스가 심화되어 가족 체계의 불균형이 오게 되면 가족 위기가 초래될 수 있다[30]. 국내에서 생체 간이식 공여자가 수혜자를 돌보는 경우도 있는데 이러한 가족원을 대상으로 수행된 질적 연구에서 이중 돌봄이라는 혹독한 일상을 가족을 지키기 위해 한다고 진술하였으며[31], 이러한 가족원들을 위하여 간병보조금 지원, 자원봉사자 활용, 돌봄 휴가 등 한국형 간이식가족 지원체계와 상담프로그램 개발을 위한 연구들이 필요할 것으로 보인다.

본 연구결과에서 생체 간이식 공여자의 간절제술은 좌측 보다 우측 간절제술을 주로 이용한 것으로 나타났는데, 초기 생체 간 이식수술에서는 공여자의 간 좌엽을 절제하였으나 점차로 간 우엽을 절제하는 것으로 변화됨으로써 생체 간이식 공여자의 안전성에 대해 더욱 관심을 가지게 되었다[16]. 일반적으로 생체 간이식 공여자의 간을 절제시 잔여 간용적이 30% 미만일 경우 공여자의 생명을 위협할 수 있는 심각한 간부전으로 이어질 수 있고, 고빌리루빈혈증 등 여러 합병증이 발생할 가능성이 높기 때문에 생체 간이식 공여자에게 30% 이상 충분한 잔여 간용적을 남기는 것이 수술 후 합병증을 감소시키기 위해 필요하다[32]. 최근에는 단순히 용적으로만 계산하지 않고 컴퓨터 단층촬영을 이용하여 가상의 선으로 간 용적을 측정함과 동시에 잔여 간이 지방간인지 확인하기 위해 간섬유스캔과 간절제술 수술실 현장에서 간생검을 통해 동결절편검사를 하여 최종확인하고 간절제술을 함으로써 생체 간이식 공여자의 용적뿐 아니라 실제 간 기능을 안전히 확보하는 방법을 사용한다[22]. 생체 간이식 공여자의 간절제술이 침습적이므로 수술 후 신체적 적응에 영향을 줄 수 있음에도 불구하고 본 연구에서 선정된 일부 논문에서는 대상자들의 간절제술 절제영역에 대해 제시하지 않았다. 따라서 향후 생체 간이식 공여자를 대상으로 수행되는 연구에서 간절제술 절제영역에 대해 명확히 확인하는 것이 생체 간이식 공여자의 수술 후 적응을 이해하는데 필요할 것이다.

최종 선정된 11개 논문에서 나타난 생체 간이식 공여자의 수술 후 적응과정에서 가장 많이 나타난 내용은 수술 전에 기대했던 것과 수술 후 실제 경험에 차이가 있다는 것이었고, 그중 예상보다 극심한 통증을 호소하였다. 이러한 결과는 국외 연구에서 생체 간이식 공여자의 41%가 수술 후 통증이 예상했던 정도 보다 너무 컸다고 하여 수술 전 교육에서 통증과 적응과정에 대한 설명이 충분하지 않았다고 불만을 나타낸 결과와 유사하다[3]. 다른 국외 연구에서 생체 간이식 공여자의 67%가 극심한 통증 때문에 다시는 간 공여를 하지 않겠다고 불만을 나타낸 바 있다[8]. 또한 신장이식 공여자를 대상으로 한 국내 연구에서도 통증으로 몸이 예전 같지 않았고 몸이 회복하는 과정에 대한 자세한 설명을 듣지 못했기 때문에 통증 등을 경험할 때 당황하였다고 하였다[30]. 생체 간이식 공여자의 통증과 관련된 연구들은 주로 개복, 복강경, 로봇 수술 유형별, 간의 절제영역별, 절개 유형별로 통증의 차이를 확인한 연구가 대부분이었고[33], 간절제술 후 통증의 생리적 기전에 관해 규명한 연구는 부족하여 이와 관련된 추후연구가 필요하다.

자발성 공여보다 비자발성 공여에서 통증의 강도를 더 높게 인지한 결과가 나타났는데[20], 이는 수술에 대한 두려움과 수술 후 일상생활을 할 수 있는지가 불명확하기 때문에 생체 간이식 공여자가 간 공여를 결정하기 어려웠다고 한 선행연구에 비추어 볼 때[11], 간 공여를 자의에 의해 선택하지 않은 공여자의 두려움이 주관적 통증점수가 높아지는 것에 영향을 주었으리라 생각해볼 수 있다. 신장이식 공여자에서도 신장을 주고 싶은 마음이 적었지만 가족의 설득으로 수동형 비자발적 의사결정을 한 경우에 통증에 대한 두려움이 큰 것으로 나타난 연구결과[34]와도 유사하였다. 따라서 간 공여를 결정한 과정을 살펴보고 이식수술 전 공여자 상담시에 개인별 맞춤형 교육으로 명확한 정보 제공을 하여 불확실성과 두려움이 저하되어야 통증 완화에도 도움이 될 수 있을 것이다. 그러나 선정된 11편 논문에서 통증에 영향을 주는 요인으로 알려져 있는 개인의 과거 통증 경험에 대한 조사가 없었다는 점은 통증과 관련된 후속 연구에서 고려해봐야 할 사항이라고 생각한다.

본 연구대상인 11편 논문에서 생체 간이식 공여자의 우울을 측정한 경우는 없었다. 국외 연구에서는 생체 간이식 공여자에서 자가보고식 우울 발생빈도가 14.9%이었고 병원에서 진단받은 경우는 4.02%로 나타났으나[9], 국내 연구에서 생체 간이식 공여자의 우울 발생률을 제시한 연구는 찾기 어려웠다. 생체 간이식 공여자의 우울 위험이 신체적 건강 인식에도 영향을 주기 때문에[35] 이식수술 전에는 정신과전문의에 의한 심리 평가가 필수로 이루어지고 있으나 이식수술 후에 공여자의 우울을 정기적으로 사정하는 경우는 많지 않았다. 국내에서 10,116명의 생체 간이식 공여자를 15년간 장기 추적하여 사망 원인을 조사한 연구에 의하면, 10년간 생체 간이식 공여자의 누적 사망률은 0.9%였고 사망자 53명 중 자살 19명, 암 9명, 교통사고 7명으로 나타나[36], 자살로 사망한 경우가 35.8%로 높은 편이므로 생체 간이식 공여자에게 지속적인 정신건강 평가와 지원이 장기간 필요함을 알 수 있다. 따라서 간이식 기증 후 1개월, 6개월, 12개월 단위로 생체 간이식 공여자의 정신건강 상태를 정기적으로 평가하는 추적 관찰 프로토콜을 표준화할 필요가 있다. 또한, 생체 간이식 공여자의 정신건강 평가 시에 회복탄력성, 외상후성장 등 심리적 지표를 같이 사정하는 것도 도움이 될 것으로 생각한다.

본 연구결과에서 생체 간이식 공여자들은 기대보다 회복되는 속도가 느려서 간이식 수술 전 자신이 하던 일에 복귀하는 기간이 예상보다 늦어졌다고 하였다. 일반적으로 의료진은 생체 간이식 공여자에게 수술 후 4~6주 지나면 집안일이나 간단한 서류업무가 가능하고[13], 간이식 수술 후 2개월이면 정상으로 회복된다고 수술 전 교육에서 설명하나[2,14] 본 연구에서 나타난 바와 같이 85% 이상의 생체 간이식 공여자들이 실제 직장에 복귀하는 기간은 6개월[10,23], 1년[19]으로 나타나 수술 전 설명과 실제로 차이가 있었다. 의사들은 객관적인 임상 자료에 의거하여 수술이 성공적이라고 얘기하지만, 환자들은 자신이 기대했던 수술 후 적응 과정과의 비교에 따라 수술이 성공적이라고 인식하기 때문에[37], 의료진은 수술 전 설명을 신중히 해야 할 필요가 있다. 수술 전 기대와 실제 경험하는 적응과정이 정확하게 일치할 수는 없지만, 수술 전에 기대했던 회복과정과 실제 수술 후 겪는 적응과정이 더디거나 악화될 경우 심리적 스트레스를 유발하므로, 예상치 않았던 결과들을 포함하여 정확하고 실제적인 정보를 제공함으로써 이러한 불일치에서 야기되는 부담감을 감소시키는 것이 필요하다[37]. 국외 연구에서 장기간 생체 간이식 공여자들을 추적 연구한 결과에서 수술 전 직업으로 돌아가는데 4년까지 소요된다고 보고하고 있고[38], 공여자의 22-48.5%가 직장으로 복귀하더라도 5~10년까지 지속적인 피로와 신체활동에서 약간의 제약 등을 경험한다고 하였다[39]. 따라서 향후 생체 간이식 공여자 대상 연구도 10년까지 장기적 추적조사를 하여 실제적으로 직장에서 정상적인 활동이 이루어지는지에 관해 조사해볼 것을 제안한다.

선정된 11개 논문 중 생체 간이식 공여자의 알코올 섭취에 조사한 것은 1편에 불과하였고 특히 3.8%가 습관성 음주로 나타났으며[23], 국외 연구에서 생체 간이식 공여자의 2%~6%에서 알코올 사용 장애가 일어난다고 보고한 결과와 비슷하다[5]. 일반적으로 알코올로 인해 간의 회복이 지연될 수 있으므로 생체 간이식 공여자의 수술 후 추후관리에서 알코올 사용 장애와 관련된 항목을 엄밀히 확인할 필요가 있다.

또한, 본 연구에서 기대하지 않았던 합병증 발생에 대해 생체 간이식 공여자의 7.3%가 예상한 것보다 상태가 좋지 않았다고 하였으며[26] 6.3%~48.3%의 합병증 발생률을 제시하였다. 이는 국외 연구에서 생체 간이식 공여자의 40%가 합병증을 경험했다는 연구[40]에 비교하면 낮은 편이다. 그러나 선정된 논문들에 나타난 합병증 발생률은 조사한 기간과 범위가 논문마다 다르기 때문에 단순하게 비교하기 어려웠다. 연구의 측정기간이 모호하다는 점과 생체 간이식 공여자의 수술 후 기간도 다양하여 범주화하기 어려우므로 향후 연구에서는 6개월~1년 기간 단위로 간이식 수술이 경과한 시점을 범주화하여 조사할 필요가 있다. 특히 합병증을 단순히 유무로 평가할 것이 아니라 수술 후 합병증의 중증도에 따라 클라비안 딘도 분류를 이용하여 체계적으로 Grade I~IV로 분류하는 것이 필요하다. Grade I 합병증은 간 효소가 정상으로 회복되지 않는 것, 병원 재원일수가 늘어나는 상황을 야기하는 합병증을 의미하고, Grade II 합병증은 약물적 치료가 필요한 것으로 예를 들어 항생제 투여가 필요한 합병증이다. Grade III 합병증은 수술, 내시경, 방사선 중재가 필요한 합병증을 의미하며, Grade IV 합병증은 생명을 위협하는 합병증이므로 중환자실 입원을 필요로 한다. Grade V는 사망에 이르는 합병증을 의미하고, 이중 Grade III, IV, V 합병증을 합쳐서 주요 합병증이라 부른다[23]. 본 연구결과에서 전체 합병증 중에서 등급별 비율을 살펴보면, Grade I 합병증 62.3%~85.0%, Grade II 합병증 3.5%~16.8%, Grade III 5%~19.4%로 나타났는데[22,23,26], 국외 연구에서는 이식수술 3개월 후 합병증 중에서 Grade I 합병증 94%, Grade II 합병증 3%, Grade III 합병증 3%로 나타나[8] 대부분 연구에서 중증도가 낮은 합병증의 비율이 높았다. 가장 흔한 합병증으로는 담도 관련 질환이 발생할 수 있으나 대부분 큰 문제없이 회복되었다[10,16]. 본 연구에서 클라비안 딘도 분류를 이용한 논문은 3편에 불구하여 선정된 논문들의 합병증을 중증도에 따라 보기에 제한점이 있으므로 향후 생체 간이식 공여자 대상의 연구를 수행할 경우에 이 분류에 의해 합병증을 분석할 필요가 있다.

또한 좌엽절제술 공여자의 합병증 발생률 보다 우엽절제술 공여자의 합병증 발생률이 더 높게 나타났으나 재원기간은 비슷하였으므로 대부분의 합병증을 안전하게 치료할 수 있었다고 보고하였고[16], 국외연구에서도 우엽절제술이 좌엽절제술에 비해 합병증 발생률은 높았으나 두 군 사이의 삶의 질은 차이가 없었다고 하여[3] 생체 간이식 공여자의 간절제술에 따른 합병증이 비교적 중증도가 낮은 단계의 합병증인 것으로 유추할 수 있다. 이러한 생체 간이식 공여자의 합병증 호소에 대해 병원에서의 검사가 정상인 경우에는 의료진이 생체 간이식 공여자가 건강염려증이나 불안 때문에 증상을 호소한다고도 생각하므로 생체 간이식 공여자의 신체적 적응에 대한 이해와 공감이 부족하다고 하였다[2]. 즉, 생체 간이식 공여자 41명의 간절제술 후 간이 재생되는 용적을 측정한 결과에서 수술 후 30일째에 수술 전 간용적의 88.5% 정도로 간이 재생되었고 간기능 검사도 정상으로 돌아왔다고 보고하여[41] 의학적 검사로는 생체 간이식 공여자의 신체적 호소들이 객관적으로 공감이 되지 않는 것이다. 따라서 이러한 생체 간이식 공여자들은 이미 생체 간이식 수술을 경험한 공여자들과의 만남 등을 통해 적응 과정에 관한 경험을 공유하고 정서적 지지를 받는 자조모임 같은 사회적 지지그룹이 필요할 것으로 생각한다[42]. 생체 간이식 공여자들이 퇴원 후 집에서 발생하는 증상 관리 등 간이식 수술 후 일상생활 관리에 대한 교육요구가 가장 높은 것으로 나타났는데, 생체 간이식 공여자들의 56.0%가 주로 인터넷 검색을 통해 정보를 얻는다고 하여 의학적으로 검증되지 않은 정보를 얻을 위험도 있었다[19]. 따라서 생체 간이식 공여자들의 수술 후 합병증 증상 등 일상생활을 관리하기 위해 국내 신뢰도가 높은 의료기관에서 정확한 자료를 토대로 동영상 교육 프로그램을 개발하여 온라인에서 생체 간이식 공여자들이 쉽게 접근할 수 있도록 하는 것이 도움이 될 것이다.

본 연구결과에서 생체 간이식 공여자가 느끼는 부담감 중 수술흉터에 대한 스트레스가 2편[10,25]에서 나타났다. 수술 절개부위가 크기 때문에 생기는 수술흉터는 복강경 간절제술이 등장하면서 좀 더 개선되었는데 Suh 등[43]의 연구에 의하면 복강경으로 인한 절개가 개복 간절제술의 역L자형 절개(inverted L incision)에 비해 생체 간이식 공여자의 만족도가 높았다. 또 Yoo 등[10]의 연구에서는 여성 생체 간이식 공여자의 수술 전 상담시에 미리 수술절개 사진을 보여줄 필요가 있다고 제안하기도 하였다. 이와 같은 국내 연구결과들과 달리 국외 연구에서는 생체 간이식 공여자가 다른 사람의 생명을 구했다는 생각으로 수술흉터를 자부심으로 생각한다고 하였다[42]. 다행히 국내 일개 병원 자료에 의하면 2015년 이후 공여자의 간절제시 복강경을 이용하는 최소한 침습수술이 증가되어 2022년에는 생체 간이식 공여자의 90%이상이 복강경 간절제술을 이용하므로[44] 생체 간이식 공여자의 수술흉터 스트레스는 감소되고 있다.

또한 생체 간이식 공여자들은 불확실한 미래와 경제적 어려움에 대한 부담을 느꼈는데[10,19,25], 국외 연구에서도 재정적 부담이 정신적 삶의 질에 부정적 영향요인이라고 보고된 바 있다[3]. 신장이식 공여자에서도 이식 수술비용과 공여 후 대부분의 의료비용이 의료보험 적용이 되지 않아 경제적 부담을 가졌다는 결과와도 일치한다[30]. 재정적 어려움이 있는 경우에는 간 공여 후 신체가 적응하는 과정에 영향을 주고[5], 경제적 부담이 낮을수록 심리사회적 적응이 높기 때문에[45] 생체 간이식 공여자의 수술 전 상담 시에 재정적 지원을 받을 수 있는 사회복지제도의 혜택에 관한 내용을 포함하여 사회경제적 어려움에 도움을 줄 필요가 있다. 특히 생체 간이식 공여자의 합병증이나 외래 추후관리에 필요한 비용 등을 마련하기 위해 장기적으로 직업 재활과 후원 등을 연결해주는 프로그램이 활성화되어야 생체 간이식 공여자들의 미래에 대해 불확실성이 감소될 것이다.

본 연구결과에서 생체 간이식 공여자들은 간 공여를 계기로 가족 관계가 깊어졌고[25], 특히 수혜자와의 친밀도가 간이식 수술 전 8.96점에서 이식수술 후 9.28점으로 상승한 것으로 나타났는데[19], 이러한 결과는 가족에게 장기를 공여한다는 의미가 수혜자와의 정서적 결합과 감사의 표현이라고 볼 수 있다는 주장과 유사한 결과이다[8]. 신장이식 수혜자는 신장 공여자를 평생의 은혜로 여김과 동시에 평생의 빚으로 여기는데 이는 한국의 문화적 특성이라고 하였고[30], 신장을 공여하는 것이 집안을 지킬 수 있는 방법으로 생각했으며 이로 인해 가족의 우애가 깊어졌다고도 하였다[29]. 그러나 국내 다른 연구에서는 생체 간이식 공여자가 가족들의 관심을 받지 못함으로써 섭섭하고 분노를 느낀다고 하였으며[14], 국외 연구에서도 간이식 수혜자를 오히려 생체 간이식 공여자가 간호해야 하는 상황이었고 간이식 수혜자는 생체 간이식 공여자에 대한 관심이 거의 없었다고 부정적 가족관계를 제시하기도 하였는데[46] 이는 각 나라마다 또는 가족마다 분위기가 다양하므로 향후 국내 다기관 대규모 연구에서 반복 연구할 필요가 있다.

본 연구에서 국내 생체 간이식 공여자의 삶의 질을 2편의 논문에서 측정했는데[10,26], 생체 간이식 공여자들의 HRQOL에서 신체적, 정신적 영역이 모두 높게 나타났으며[26], 이식수술 후 자신의 전반적 삶의 만족도에 대해 87.0%가 만족한다고 하였다[10]. 이러한 결과는 국외 연구에서 이식수술 후 공여자의 HRQOL에서 신체적 영역은 일시적으로 이식수술 후 3개월까지 저하되었다가 서서히 적응하는 것으로 나타났고 정신적 영역의 HRQOL은 이식수술 후에도 계속 안정적으로 유지되었다고 한 결과와 비슷한 맥락이었다[3,6]. 그러나 2편의 논문에서 사용한 도구가 한국인의 자가평가 건강수준 측정도구(KHP 1.0) [10]와 SF-12v2 [26]로 각각 다른 도구를 사용하였으므로 두 논문에 나타난 삶의 질을 객관적으로 비교하기 어려웠다. 또한 이러한 도구들은 생체 간이식 공여자에 적합하게 개발된 도구가 아니므로, 생체 간이식 공여자들이 간이식 수혜자의 사망이나 수혜자의 건강 상태에 따라 삶의 질에 영향을 받는다는 연구결과[10,25]를 반영하여 수혜자의 건강 상태를 포함하는 항목이 있는 민감성 높은 도구 개발이 추후연구에서 수행되기를 제안한다.

생체 간이식 공여자는 간을 기증하기로 결정한 것에 만족하는 경우는 96.8%[26], 간 기증을 후회하지 않는다는 경우가 93.5%[10]로 나타나 간이식에 대한 만족도가 높게 나타났고, 주변의 배려와 효자라는 칭송과 격려를 받으면서 덕분에 힘을 얻어 잘 견디어 내었다고 하였다[25]. 이는 신장이식 공여자에서도 친구와 동료와 이웃의 인정과 지지가 클 때 잘 적응하였다고 한 결과와도 일맥상통한다고 볼 수 있다[30]. 국외 연구에서 생체 간이식 공여자의 95%가 만약 수술 전이라면 다시 간 공여를 선택하겠다고 긍정적 응답을 한 것과도 비슷한 결과이고[47], 생체 간이식 공여자들이 간 공여 후 다른 사람의 삶을 변화시켰다는 만족감이 증진되고 자신에 대한 가치가 높아짐을 느꼈다는 것과도 일치하는 결과이다[42]. 또한 간이식 수술 후 생체 간이식 공여자의 만족도를 증가시키는 요인 중 하나가 장기이식팀과의 친밀하고 지지적인 의사소통의 영향이 컸다는 결과보고를 바탕으로[48] 간이식 수술 후에도 단순한 외래 진료가 아니라 다학제팀에서 장기이식코디네이터가 장기적으로 지속적인 관리를 할 수 있는 프로그램을 개발하는 것이 생체 간이식 공여자의 만족도를 증진시키는데 도움이 될 수 있을 것이다.

영성 수준과 종교는 생체 간이식 공여자의 삶의 만족도를 증진시키는데 중요한 지지를 하는 원천이 된다고 하였으나[40], 본 연구에서 선정된 국내 연구 11편에서 종교나 영성과의 관련성을 언급한 경우는 없었기에 추후 이와 관련된 연구가 필요할 것으로 생각한다.

이상과 같이 본 연구에서 고찰한 내용을 통합하면 생체 간이식 공여자의 수술 후 적응은 다음과 같다. 첫째, 생체 간이식 공여자들은 수술 전 설명으로 들었던 통증, 적응속도, 합병증에 대한 기대와 수술 후 실제 경험에서 차이를 인식하여 신체적 적응에 어려움이 있었으며, 따라서 현실적인 정보를 제공하는 수술 전 상담과 교육 개발이 필요하다. 둘째, 생체 간이식 공여자들은 수술 후 생긴 흉터, 불확실한 미래와 재정적 부담으로 심리적 부담감을 가지게 되었으나, 최근 대부분의 생체 간이식 공여자들이 복강경 간절제술을 하기 때문에 흉터에 대한 부담은 사라졌고 이식과 관련된 재정적 부담은 사회복지제도와 후원의 활성화, 직업재활의 연계로 사회적 지원이 필요하다. 셋째, 생체 간이식 공여자들은 이식수술 후 깊어진 가족관계와 삶의 만족감이 증진되었다. 그러나 생체 간이식 공여자의 건강관련 삶의 질을 측정하는 도구가 생체 간이식 공여자에게 적용하기에는 민감도가 높지 않아 추후 장기이식 공여자를 위한 삶의 질 측정 도구 개발과 표준화가 이루어져야 할 것이다.

본 연구의 간호학적 의의는 생체 간이식 공여자의 수술 후 신체적, 심리사회적 적응에 관한 연구들을 고찰한 연구가 국내에 없는 상황에서 국내 생체 간이식 공여자의 수술 후 적응을 분석하여 통합적으로 고찰함으로써 간이식 수혜자뿐 아니라 생체 간이식 공여자의 수술 후 적응에 대한 관심을 증진시켜 간호 대상자의 범위와 역할을 확대해야 하는 방향성을 제시하였다는 것이다.

본 연구는 다음과 같은 제한점이 있으므로 결과 해석에 주의가 필요하다. 첫째, 본 연구에서 논문의 선정과정 중 질 평가단계에서 외부 전문가 1인의 자문을 받아 신뢰도를 높이려고 노력하였으나 검색단계와 분석단계를 단독 연구자가 수행하였기에 잠재적 주관성을 배제하기 어렵다. 둘째, 최종 포함된 국내 연구 11편 중 질적 연구가 1편[25]에 불과하여 간 공여 후 공여자들의 생생한 긍정적, 부정적 적응 경험에 대해 추가적으로 파악하기 어려웠다. 셋째, 다기관 대규모 코호트 연구 1편[23]을 제외한 10편의 연구가 특정한 일개 기관에서 생체 간이식 수술을 받은 공여자를 대상으로 수행되었기 때문에 일반화에는 신중을 기할 필요가 있다. 넷째, 동료평가를 거쳐 학술지에 게재된 논문만을 연구에 포함하였으므로 생체 간이식 공여자의 수술 후 적응과 관련된 연구이지만 학술지에 게재되지 않아 본 연구에 반영되지 못한 문헌이 존재할 수 있다.

## 결론 및 제언

본 연구에서는 국내 생체 간이식 공여자들을 대상으로 수술 후 신체적, 심리사회적 적응에 대해 통합적으로 파악하였다. 최종 포함된 11편의 논문을 통해 국내 생체 간이식 공여자들의 신체적 적응 과정에서 기대와 현실 차이에서 오는 신체적 부담, 수술 후 생긴 흉터와 불확실한 미래와 재정적 힘듦에서 오는 심리적 부담감으로 이식수술 후 신체적, 심리사회적 적응에 부정적인 측면이 보이지만, 가족에게 간을 공여함으로써 가족관계가 깊어지고 삶에 대한 만족이 증진되어 간이식 수술 후 적응에 긍정적인 영향이 미칠 수 있음을 시사하고 있다. 특히 본 연구결과를 통해 생체 간이식 공여자의 신체적 적응에 관한 현실적인 정보를 반영한 상담 프로그램 개발의 필요성이 제기된다.

본 연구에서는 다음과 같은 제언을 하고자 한다. 첫째, 생체 간이식 공여자에게 적합한 민감도 높은 삶의질 측정 도구의 개발을 제언한다. 둘째, 대부분의 논문들이 일개 기관의 생체 간이식 공여자를 대상으로 이루어졌으므로 향후 다기관에서 대규모 코호트연구로 생체 간이식 공여자의 신체적, 심리사회적 적응과 영향요인에 관해 탐색하는 연구의 필요성을 제언한다.

## Figures and Tables

**Table 1.** Quality Appraisal of Retrieved Studies (N = 12)

Author (year)	Study design	Quality criteria						
		S1	S2	4.1	4.2	4.3	4.4	4.5
Jo et al. [16]	Quantitative	Yes	Yes	Yes	Yes	Yes	Yes	Can’t tell
Lee et al. [17]	descriptive	Yes	Yes	Yes	Yes	Yes	Yes	Yes
Yoo et al. [10]	study	Yes	Yes	Yes	Yes	Yes	Yes	Yes
Chon et al. [18]		Yes	Yes	Yes	Yes	Yes	Yes	Yes
Kim et al. [19]		Yes	Yes	Yes	Yes	Yes	No	Yes
Bea et al. [20]		Yes	Yes	Yes	Yes	Yes	Yes	Yes
You et al. [21]		Yes	Yes	Yes	No	Yes	Yes	No
Lee et al. [22]		Yes	Yes	Yes	Yes	Yes	Yes	Can’t tell
Lee et al. [23]		Yes	Yes	Yes	Yes	Yes	Yes	Yes
Jung & Bang [24]		Yes	Yes	Yes	Yes	Yes	Yes	Yes
Lee et al. [26]		Yes	Yes	Yes	Yes	Yes	Yes	Yes
		S1	S2	1.1	1.2	1.3	1.4	1.5
Bang et al. [25]	Qualitative study	Yes	Yes	Yes	Yes	Yes	Yes	Yes

S1: Are there clear research questions?; S2: Do the collected data allow to address the research questions?. 1.1: Is the qualitative approach appropriate to answer the research question? 1.2: Are the qualitative data collection methods adequate to address the research question? 1.3: Are the findings adequately derived from the data? 1.4: Is the interpretation of results sufficiently substantiated by data? 1.5: Is there coherence between qualitative data sources, collection, analysis and interpretation? 4.1: Is the sampling strategy relevant to address the research question? 4.2: Is the sample representative of the target population? 4.3: Are the measurements appropriate? 4.4: Is the risk of nonresponse bias low? 4.5: Is the statistical analysis appropriate to answer the research question?

**Table 2.** The Characteristics and Findings of the Studies Included in the Integrative Review (N = 11)

Study ID	Author (year)	Study design	Sample characteristics	Research methods	Main findings
A1	Jo et al. [16] (2001)	Descriptive study	*N = 104	Retrospective medical record review	*Length of hospital stay: 17.1 days (left lobectomy), 18.0 days (right lobectomy)
			-Men 84 (80.8%), Women 20 (19.2%)		*Right lobectomy showed longer intensive care unit (ICU) stays and recovery times for liver function, but discharge timing remained consistent across both groups.
			-Age: median 28 years (range 16–54 years)		*Hyperbilirubinemia: 8 cases in the right lobectomy; all normalized within 14 days.
			*Type of lobectomy		*Complications: bile leakage, bleeding
			-Left lobectomy 50 (48.1%)		-Right lobectomy: 7 cases (13.0%)
			-Right lobectomy 54 (51.9%)		-Left lobectomy: 2 cases (4.0%)
			*Relationship to donors		-All complications were successfully treated.
			-Child 35 (33.6%)		
			-Sibling 19 (18.3%)		
			-Spouse 9 (8.7%)		
			-Non-blood relative 25 (24.0%)		
			-Non-relative 15 (14.4%)		
A2	Lee et al. [17] (2002)	Descriptive study	*N = 386	Retrospective medical record review	*Out of the donors, 368 (95.8%) returned to normal liver function (as shown by LFT results) within 2 weeks post-transplantation. An additional 6 donors recovered by 4 weeks, and the remaining 12 donors reached normal levels after 3 months.
			-Men 297 (76.9%), Women 89 (23.1%)		*A total of 56 complications occurred in 52 patients (13.5%).
			-Age: mean 31 years (range 17–47 years)		Complications occurring within 4 weeks postoperatively: 46 cases
			*Type of hepatectomy		-Biliary complications: 18
			-Left hepatectomy 167 (43.3%)		-Pleural effusion: 9
			-Right hepatectomy 219 (56.7%)		-Intra-abdominal abscess: 5
					-Vascular complications: 8
					Complications occurring after 4 weeks postoperatively: 10 cases
					-Biliary complications: 4
					*Interventional treatment: 29 cases
A3	Yoo et al. [10] (2004)	Descriptive study	*N = 84	Prospective study	*Length of hospital stay: mean 11.4 days
			-n=46 (Living liver donor who is more than 4 months postoperatively): Men 29 (63.0%), Women 17 (37.0%)		*LFT results remained within the normal range for one year, with the exception of 3 cases.
			-n=42 (Control normal group)		*Postoperative complications:
			-Age: mean 35.3 years		-Inpatient period: 10.9%
			*Relationship to donors		-Within 4 months post-surgery: 6.5%
			-Offspring 17 (37.0%)		: 5 cases of gastrointestinal problems and abdominal pain.
			-Parent 12 (26.1%)		-Surgical comparison: No significant differences were found based on the specific type of hepatectomy
			-Spouse 6 (13.0%)		*Return to work: 76.0% returned to work within 3 months.
			-Relative 3 (6.5%)		*Psychosocial outcomes
			-Nonrelatives 4 (8.7%)		-95.6% felt a sense of reward from the liver donation.
			*Type of hepatectomy		-Regret due to recipient’s death: 6.5% (recipient mortality rate 13.0%).
			-Right lobectomy 31 (67.4%)		-87.0% are currently satisfied with their life post-transplant.
			-Left/left lateral lobectomy 15 (32.6%)		-59.5% experienced psychological distress related to the surgical wound/scarring.
					-A financial burden was reported by 37% of the participants.
					*Health profile analysis: Korean Health Profile 1.0 (KHP 1.0)
					-Physical role functioning was limited, emotional health scores were higher than those of the control group.
A4	Chon et al. [18] (2005)	Descriptive study	*N = 91	Cross-sectional survey	*Length of hospital stay: < 20 days 82.4%, ≥ 20 days 17.6%
			-Men 61 (67.0%), Women 30 (33.0%)		*Complication-related readmission: 8.8%
			-Age: mean 33.0 years		*Recovery period: 2～41 months
			*Relationship to donor		*64.8% of donors would recommend liver donation to others, which was associated with the recipient's survival rate (87.0%).
			-Child 44 (48.3%)		*Preferred source of information on liver donation: coordinator 55.0%, physician 24.1%
			-Sibling 15 (16.5%)		*The Mishel Uncertainty in Illness Scale
			-Spouse 11 (12.1%)		-Mean 51.5
			-Others 21 (23.1%)		-A longer hospital stay (over 20 days) and readmission due to complications were associated with higher levels of uncertainty.
			*Time since transplantation		
			-≤ 12 months 38.4%		
			-13–24 months 24.2%		
			-≥ 25 months 37.4%		
A5	Kim et al. [19] (2007)	Descriptive study	*N = 103	Cross-sectional survey	*Time to return to work: <1 month 16.5%, 1~3 months 30.9%, 3~6 months 29.9%, 6 months~1 year 19.6%, 1~2 years 3.1%
			-Men 74 (71.8%), Women 29 (28.2%)		*Little to no restriction in work life 75.0%
			-Age: mean 34.4years		*Perceived economic status: extreme financial hardship 8.8%, somewhat insufficient 32.4%
			*Relationship to donor		*Physical discomfort necessitating medical attention or medication 22.5% (digestive disorder 11, surgical wound 10, fatigue 9)
			-Child 48.0%		*Intimacy with the recipient: Increased from 8.96 before the liver donation to 9.28.
			-Parent 16.7%		*Knowledge level: mean 2.19 (Knowledge of the "recipient's surgical methods and success rates" was high, whereas knowledge of "preventive measures for post-donation complications" and "symptom management after liver donation" was low.)
			-Spouse 11.8%		*Educational needs: mean 3.47 (The need for "postoperative care after liver donation" was the highest, while "intake of herbal medicine after liver donation" was the lowest.)
			-Sibling 11.8%		*A significant correlation between the level of knowledge and the duration of return to work.
			-Relatives 8.8%		
			-Non-Relatives 2.9%		
			*Time since transplantation		
			-Mean 35.81 months		
			-≤ 12 months 22.0%		
			-13–84 months 75.0%		
			-≥ 85 months 3.0%		
A6	Bea et al. [20] (2009)	Descriptive study	*N = 64	Cross-sectional survey	*According to the motives for liver donation
			-Men 50 (78.1%), Women 14 (21.9%)		-Volunteer donor n=48
			-Age		-Non-volunteer donor n=32
			-≤ 29 years 35 (54.7%)		*Non-volunteer donors reported higher pain scores compared to volunteer donors.
			-30–39 years 13 (20.4%)		*Subjective pain scores (5~8 points) measured by the visual analog scale were higher than those of patients undergoing liver resection for hepatocellular carcinoma (2~3 points).
			-≥ 40 years 16 (24.9%)		*There was a significant positive correlation between preoperative state anxiety and objective pain scores measured by behavioral observations.
			*Relationship to donor		
			-Sons and daughters 53.1%		
			-Family in law 15.6%		
			-Brothers and sisters 12.5%		
			-Parents 9.4%		
			-Spouse 6.3%		
			-Pure donation 3.1%		
			*Type of hepatectomy		
			-Right hepatectomy 81.3%		
			-Left hepatectomy 18.7%		
A7	Lee et al. [22] (2014)	Descriptive study	*N = 245	Retrospective review of the medical records	*Length of hospital stay: mean 12.79 days
			-Men 173 (70.6%), Women 72 (29.4%)		*Postoperative complications (Clavien-Dindo classification): 46.1%
			-Age: mean 30.74 years (16–59 years)		-Class I 96 (39.2%): wound related problem 53
			*Right lobectomy: 100%		-Class II 4 (1.6%): decreased hemoglobin 2
					-Class IIIA (requiring intervention), 10 (4.1%): 3 cases of wound infection, 3 cases of biloma.
					-Class IIIB (requiring reoperation), 3 (1.2%): 1 case of small bowel perforation.
A8	Lee et al. [23] (2017)	Descriptive study	*N = 832	Prospective survey	*Length of hospital stay: mean 10.35 days
			*Men 553 (66.5%), Women 279 (33.5%)		*Complication rates did not differ between left lobectomy and right lobectomy․
			*Age: mean 33.9 years		*Overall complication rate: 9.3%
			*Type of hepatectomy		-Biliary complication rate: 1.7%
			-Left lobectomy 59 (7.1%)		-Clavien-Dindo grade ≥ III: 1.9%
			-Right lobectomy 773 (92.9%)		*All donors returned to normal activity and recovered normal or near normal liver function within 6 months of lobectomy.
			*Relationship to donor		*At six months post-donation, ALT levels were significantly higher in donors with an RLV ≤ 30% compared to those with an RLV > 30%.
			-Left lobectomy child 40.7%		*Graft volume: left lobectomy 404.4g (RLV 60.1%), right lobectomy 729.4 g (RLV 36.2%)
			-Right lobectomy child 72.2%		*Alcohol consumption: none 54.4%, social drinking 41.8%, habitual drinking 3.8%
A9	Jung & Bang [24] (2018)	Descriptive study	*N = 91	Retrospective review of the medical records	*Length of hospital stay: mean 9.24 days
			-Men 54 (59.3%), Women 37 (40.7%)		*Operation method
			-Age: mean 35.68 years (17–61 years)		-Laparoscopy 71 (78.0%), open 20 (22.0%)
			*Relationship to donor		*Incision
			-Offspring 54 (59.4%)		-Right lobe 83 (91.2%)
			-Spouse 18 (19.8%)		-Left lobe 6 (6.6%)
			-Sibling 10 (11.0%)		-Others 2 (2.2%)
			-Relatives 5 (5.5%)		*Complication rates: 40 cases (44.0%)
			-Parent 4 (4.4%)		*There was no significant difference in pain scores between laparoscopic hepatectomy and open hepatectomy. However, a significant difference was observed in the length of hospital stay.
					*Shorter hospital stays were significantly associated with the absence of complications, pain management using patient-controlled analgesia alone, and the use of the laparoscopic approach.
A10	Bang et al. [25] (2021)	Phenomenological study	*N = 8	Colaizzi’s method of data analysis on interview data	*Five theme clusters emerged:
			-Men 6 (75.0%), Women 2 (25.0%)		*A painful decision for liver donation: A desperate choice to save parents at the threshold of life and death. The triple burden of physical, psychological, and financial hardships during donation preparation.
			-Age: mean 26.4 years (21–34years)		*The agony of both mind and body that overpowers youth. Enduring excruciating postoperative pain and the lasting presence of surgical scars.
			*Time since donation: mean 23.1 months		*The bitter and bare face of reality encountered by a young donor. Coping with postoperative complications and diminished physical strength. An uncertain future shadowed by the recipient’s deteriorating health.
					*Feeling the power of love that fills the space of the removed organ. Enduring through the consideration and recognition of others. Strengthening resilience through the precious bonds of family.
					*Liver donation becoming priming water for maturity. Discovering the worth of life and achieving spiritual maturity through sacrifice.
A11	Lee et al. [26] (2022)	Descriptive study	*N = 124	Cross-sectional survey	*Length of hospital stay: mean 9.5 days
			-Men 70 (56.5%), Women 54 (43.5%)		*Recipient mortality rate 8.9%
			-Age: mean 37.9 years (range 19–63years)		*Complications were observed in 60 subjects (48.3%) according to the Clavien-Dindo classification (Grades I–III): Grade I 40.3%, Grade II 5.6%, Grade III 2.4%.
			*Relationship to donor		*Health-related quality of life (HRQOL) was assessed using the Korean version of the 12-item Short Form Health Survey version 2 (SF-12v2). The mean scores were 51.48±7.44 for the physical component summary and 52.97±8.47 for the mental component summary.
			-Children 71.0%		*Most participants (96.8%) reported satisfaction with the donation, showing a correspondingly high level of HRQOL
			-Spouse 13.7%		*The areas where subjects experienced outcomes "worse than expected" were pain (34.7%), slow recovery speed (22.6%), prolonged hospital stay (9.7%), and complications (7.3%).
			-Sibling 8.9%		
			-Parent 6.5%		
			*Time since donation: mean 2.1 years		
			-< 1 year 34 (27.4%)		
			-1–5 years 79 (63.7%)		
			-5–10 years 9 (7.3%)		
			-> 10 years 2 (1.6%)		

LFT = Liver function testing; HRQOL = Health-related quality of life; RLV = Remnant liver volume; ALT = Alanine aminotransferase.

## Appendix 1. List of studies included in an integrative review

A1. Jo SH, Hwang S, Lee SG, Park KM, Lee YJ, Ahn CS. Safety of donor in adult-to-adult living donor liver transplantation. Journal of the Korean Surgical Society. 2001;60(3):314-319.

A2. Lee IS, Lee HJ, Ryoo MS, Ko GY. Postoperative complications in liver transplantation donors and usefulness of interventional management. Journal of the Korean Radiological Technologist Association. 2002;28(1):83-89.

A3. Yoo JY, Yi NJ, Suh KS, Kwon CH, Choi SH, Lee KU. Donor quality of life in living donor liver transplantation. The Journal of the Korean Society for Transplantation. 2004;18:73-80.

A4. Chon HO, Park HR, Park JH. Uncertainty and factors affecting organ donation in living liver donors. Journal of Korean Public Health Nursing. 2005;19(1):129-138.

A5. Kim EM, Byun NI, Kim KS, Bea SS, Kim MA. A study on the knowledge and educational needs of living liver donors. Clinical Nursing Research. 2007;13(2):51-60.

A6. Bea SS, Lee HY, Lee KH. Preoperative anxiety and postoperative pain related to donation spontaneity in living donors undergoing liver transplantation. Journal of East-West Nursing Research. 2009;15(2):82-90.

A7. Lee JG, Han DH, Choi SH, Choi GH, Choi JS. Surgical outcomes and complications after right hepatectomy in living donation for adult liver transplantation: single center experiences from 245 cases. Journal of Korean Society of Transplantation. 2014;28:19-24. http://doi.org/10.4285/jkstn.2014.28.1.19

A8. Lee JG, Lee KW, Kwon CHD, Chu CW, Kim BW, Choi DL. et al. Donor safety in living donor liver transplantation: The Korean organ transplantation registry study. Liver Transplantation. 2017;23:999–1006. https://doi.org/10.1002/lt.24778

A9. Jung JH, Bang KS. Factors affecting the postoperative pain and length of hospital stay of liver transplantation donors. The Journal of Korean Academic Society of Nursing Education. 2018;24(4):433-442. https://doi.org/10.5977/jkasne.2018.24.4.433

A10. Bang MS, Shin HY, Ryu M, Kwon SH. Young adult donor’s experiences of living donor liver transplantation. Journal of Korean Academy of Nursing. 2021;51(1):105-118. https://doi.org/10.4040/jkan.20235

A11. Lee YS, Koh CK, Yi NJ, Suh KS, Lee KW. Does living liver donors’ underestimation about surgical outcomes impact on their health-related quality of life after donation?: a descriptive cross-sectional study. Health and Quality of Life Outcomes. 2022;20:146. https://doi.org/10.1186/s12955-022-02055-0
